# Lignocellulosic Bioethanol and Biobutanol as a Biocomponent for Diesel Fuel

**DOI:** 10.3390/ma14195597

**Published:** 2021-09-26

**Authors:** Michal Obergruber, Vladimír Hönig, Jan Jenčík, Jiří Hájek, Dominik Schlehöfer, Tomáš Herink

**Affiliations:** 1 Department of Chemistry, Faculty of Agrobiology, Food and Natural Resources, Czech University of Life Sciences Prague, Kamýcká 129, 169 21 Prague, Czech Republic; obergruber@af.czu.cz (M.O.); jencikj@af.czu.cz (J.J.); jirihajek@af.czu.cz (J.H.); 2 ORLEN UniCRE a.s., Záluží 1, 436 70 Litvínov, Czech Republic; dominik.schlehofer@orlenunicre.cz (D.S.); tomas.herink@unipetrol.cz (T.H.)

**Keywords:** butanol, ethanol, biomaterials, alternative fuel, second generation, distillation, cetane number, lubricity, CFPP

## Abstract

In this paper, the fuel properties of mixtures of diesel fuel and ethanol and diesel fuel and butanol in the ratio of 2.5% to 30% were investigated. The physicochemical properties of the blends such as the cetane number, cetane index, density, flash point, kinematic viscosity, lubricity, CFPP, and distillation characteristics were measured, and the effect on fuel properties was evaluated. These properties were compared with the current EN 590+A1 standard to evaluate the suitability of the blends for use in unmodified engines. The alcohols were found to be a suitable bio-component diesel fuel additive. For most physicochemical properties, butanol was found to have more suitable properties than ethanol when used in diesel engines. The results show that for some properties, a butanol–diesel fuel mixture can be mixed up to a ratio of 15%. Other properties would meet the standard by a suitable choice of base diesel.

## 1. Introduction

Renewable fuels are increasing in the fuel mix every year. The increasing share is driven by economic and political factors as well as oil scarcity. Renewable fuels are seen as one of the options to reduce dependence on oil and contribute to reducing emissions from internal combustion engines [1].

Diesel engines produce different types of emissions. Like petrol engines, they produce CO_2_, CO, NO_x_, and unburned hydrocarbons (UHC). In addition, they also emit particulate matter (PM_2.5_) of various sizes and compositions [2]. Based on available data on health effects, these PMs can cause serious health problems. Exposition can cause pulmonary fibrosis [3], lung cancer [4], asthma [5], or DNA mutation [6,7].

Reducing the amount of particulate matter emitted is therefore a major initiative that has strong support from policy makers around the world [8]. The emissions can be lowered by installing filters [9] or by the addition of other compounds that do not produce PM, such as alcohols, including ethanol or butanol [10], or has overall lower emission levels, such as biodiesel (fatty acid methyl ester—FAME) [11].

Yet, biodiesel is currently the most widely used alternative fuel. Currently, the most widely used biodiesel production process is transesterification, which is a chemical reaction between oil or vegetable or animal fat (triglycerides) and alcohol in the presence of a catalyst to produce ethyl esters or methyl esters (biodiesel) and glycerol (byproduct). There are many types of catalytic processes, including alkali-catalyzed transesterification, acid-catalyzed transesterification, acid- and alkali-catalyzed two-step transesterification, enzyme-catalyzed transesterification, or non-catalytic conversion techniques for transesterification, which further determine the quality characteristics of the fuel [12]. 

The main raw materials for the current production of biodiesel are vegetable oils (olive, sunflower seed, corn, peanut, soybean, rapeseed, corn, palm, etc.) [13], or algae and microalgae (*Chlorella* sp., *Chlamydomonas reinhardtii*, *Dunaliella tertiolecta*, *Bacillariophyceae*, *Chlorophyceae*, *Chrysophyceae*, etc.) [14,15]. Recently, the use of waste cooking (frying) oils has gained much attention as an alternative, environmentally friendly, abundant, and sustainable feedstock for biodiesel production, due to their lower cost, compared to other feedstocks, and the elimination of the negative harmful environmental impacts of dumping waste oils from households and industry down the drain [16].

Because of the above problems with biodiesel, scientists are focusing more on other biofuels that can also be produced from waste and other non-edible materials, such as alcohols. The conventional way to produce alcohol is by chemically converting simple sugars from grains, such as corn or sugar cane. This type of fuel is considered a first generation biofuel [17,18]. A more sustainable method is to use lignocellulosic materials, i.e., materials composed of cellulose, hemicellulose, and lignin. Biofuels made from these materials are considered to be second generation biofuels.

**Figure 1:**
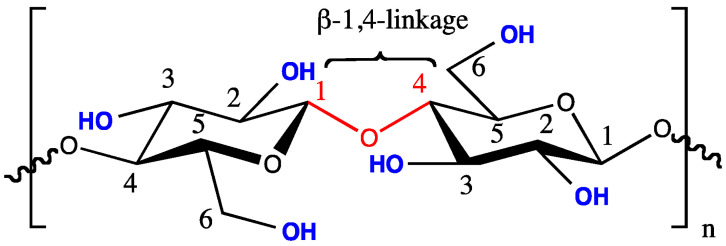
Chemical representation of a single cellulose chain repeat unit depicting two glucose units with a β-1,4-glycosidic linkage [22].

Cellulose, shown in Figure 1, is the main component of biomass (about 45% of the dry weight of wood) and consists of a D-glucose polymer linked by a β-1,4 glycosidic bond to form cellobiose molecules [19]. These polymer chains are linked by hydrogen bonds and van der Waals forces in which the glucose unit is compactly bound to the others to form so-called cellulose fibers. These are wrapped with hemicellulose [20]. 

The crystallinity of cellulose depends on this ordered structure of cellulose fibers. The strong interaction of hydrogen bonds between the cellulose fibers makes it insoluble in water but soluble in dilute acid solutions at high temperature [19,21].

**Figure 2:**
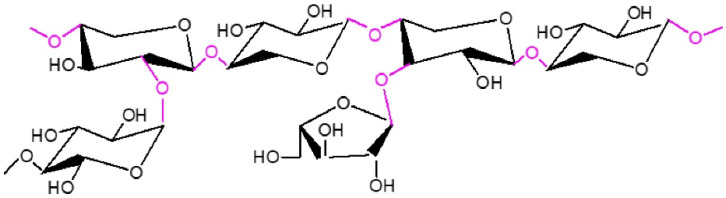
Chemical representation of a hemicellulose chain consisting of xylose and arabinose pentoses [23] (edited).

Hemicellulose, shown in Figure 2, is a copolymer composed of various pentoses, hexoses, and uronic acids. Common sugars contained in hemicellulose are xylose, arabinose, mannose, or galactose with 50–200 units. The main component of agricultural biomass and hardwood hemicellulose is xylan, while the main component of softwood is glucomannan [17]. Due to the presence of pentose sugars, hemicellulose has an affinity for water, and at high concentrations, aqueous solutions are viscous [19,21].

Lignin is a copolymer of cross-linked polymers of phenolic monomers. i.e., coumaryl alcohol, sinapyl alcohol, and coniferyl alcohol. These phenolic monomers are linked to each other by carbon–carbon (C–C) and carbon–ether (C–O) bonds [24]. This type of binding increases plant cell wall stability and resistance to pathogenic infections [25]. It acts as a physical barrier against enzymatic hydrolysis and microbial decomposition because it is tightly bound to cellulose fibers [24]. Enzymatic or microbial delignification is difficult because lignin derivatives act as toxic compounds for microorganisms and reduce the activity of hydrolytic enzymes. The location of lignin is between cellulose and hemicellulose, which bind to each other [21,24]. 

Different lignocellulosic sources have different chemical compositions. Table 1 shows common examples of lignocellulosic materials with approximate proportions of the different biopolymers [26].

The complex structure of lignocellulosic material makes the processing of fermentable sugars difficult. Prior to fermentation, the tight structure of the plant biomass must be disrupted to make it amenable to enzymatic hydrolysis, which is done by various types of pretreatment [27,28].

Pretreatment of lignocellulose can be carried out by physical, chemical, physicochemical, and biological agents. Physical pretreatment includes mechanical interactions and irradiation [29]. Chemical pretreatment includes acid or alkaline pretreatment, treatment with ionic liquids, organic solvents, the use of sulphites, alkaline wet oxidation or ozonation [30]. Physicochemical pre-treatment includes steam explosion (catalyzed or uncatalyzed), hot water pre-treatment, ammonia fibers explosion or carbon dioxide explosion [31]. Biological agents include the use of microorganisms to pre-treat lignocellulose. Combinations of individual pretreatments that target specific chemical components of lignocellulose have proven to be a promising path [32].

Pre-treatment is followed by hydrolysis and fermentation, which can take place separately or simultaneously. During hydrolysis, polysaccharides are broken down into simple sugars. This process may take place in the presence of acids, enzymes or both simultaneously [33].

The success of fermentation depends on the quality of the pretreatment. In addition, parameters such as temperature, pH, agitation or oxygen concentration can cause undesirable effects on the metabolism of microorganisms [34].

Microorganisms that are able to ferment pentose or hexose sugars to alcohols are, for example, *Clostridium acetobutylicum*, *Klebsiella pneumoniae*, *Leuconostoc mesenteroides*, *Sarcina ventriculi*, or *Zymomonas mobilis*. Some species of fungi are also capable of fermentation to form alcohols, including *Aspergillus oryzae*, *Endomyces lactis*, *Kloeckera sp.*, *Kluyreromyees fragilis*, *Mucor sp.*, *Neurospora crassa*, *Rhizopus sp.*, *Saccharomyces beticus*, *S. cerevisiae*, *S. elltpsoideus*, *S. oviformis*, *S. saki*, or *Trulaporium cutaneum* [35].

Due to the aforementioned feedstock implications and subsequent processes that require extensive and costly chemical or physical pretreatment, this has proven to be a major impediment to large-scale fuel production [18,36,37].

The most common biocomponent in diesel fuel in the European Union is a biodiesel. Despite the advantages of using biodiesel, one of the main issues concerning the use of biodiesel is its poor low-temperature flow property, and many researchers have found that adding biodiesel to diesel fuel will increase NO_x_ emissions [38,39,40,41,42,43,44]. For this reason, using the same bioalcohols used in a gasoline engine can be an interesting option. Table 2 shows the fuel properties of winter diesel fuel Class F, FAME Class F, ethanol, and n-butanol.

In addition to suitable fuel properties, it has also been shown that the use of these alcohol blends can suppress soot formation (problem of pure diesel fuel) without significantly increasing NO_x_ emissions (problem of FAME), eliminating the smoke–NO_x_ trade-off [45,46,47].

**Table 2:** Comparison of the properties of butanol isomers with other conventional fuels [48,49,50,51,52,53,54,55,56,57,58,59].

Properties	Diesel Fuel (Class F)	FAME (Class F)	Ethanol	n-Butanol
Molecular weight	198.4	242–294	46.07	74.11
Cetane number	>49	>51	5–8	12
Research octane number	20–30	—	108	94
Motor octane number	—	860–900	89–103	78
Density [kg/m^3^] at 20 °C	820–860	>101	789	808
Flash point [°C]	>55	<−20	14	35
CFPP [°C]	<−20	—	<−51	<−51
Cloud point [°C]	−10 to −34	—	—	—
Lubricity WSD [µm]	<460	427–671	1057	607
Water solubility at 25 °C [g/L]	<0.2	<0.5	miscible	73
Boiling point [°C]	180–370	295–366	78.5	117.7
Flammability [vol%]	0.6–7.5	—	3.3–19	1.4–11.2
Reid vapor pressure [kPa]	0.2–0.7	0.2–0.6	16.5	6
Viscosity [mm^2^/s] at 25 °C	2–4.5	3.5–5	1.07	2.63
Energy density [MJ/L]	35.86	32.7	25	29.2

Ethanol–diesel fuel blends are commonly used in some countries, sold under commercial names, such as E-diesel (containing about 7–15% ethanol), or O2Diesel™ (consisting of 7.7% vol. ethanol), among others [60]. However, the engine usually has to be modified for these blends. 

This article aims to answer what is the maximum permissible level of ethanol and butanol in diesel fuel to ensure that the mixture can be used for unchanged engines, i.e., to meet the EN 590+A1 standard, and also, which alcohol–diesel blend has better fuel properties. This analysis will provide a comprehensive and practical view of these fuel blends in terms of everyday usability. 

## 2. Materials and Methods

To determine the effect of n-butanol and ethanol in diesel fuel, mixtures with working names were selected as follows:

- ETH *x*: vol% ethanol and (100 − *x*) vol% diesel fuel (e.g., ETH 5).
- BUT *x*: vol% n-butanol and (100 − *x*) vol% diesel fuel (e.g., BUT 5).

Pure diesel fuel was used for the measurements. It complies with the standard EN 590 class F—winter without FAME content (produced by Čepro, a.s.). The water content was 105 mg.kg^−1^, and the oxidative stability exceeded 20 h. n-Butanol AR (Analytical Reagent purity) was produced by LachNer, s.r.o. The tested bioethanol for comparison fully complied with the requirements of EN 15376:2014 standard.

To identify the fuel properties of the mixtures, the physiochemical properties were determined. In the evaluation of the fuel, the cetane number, cetane index, density, flash point, kinematic viscosity, lubricity, cold filter plugging point, cloud point, and distillation characteristics were measured. These fuel properties were compared with fuels containing different volumetric amounts of alcohols. 

An analytical method for the determination of ethanol and n-butanol in diesel fuel using gas chromatography with flame-ionization detection (GC-FID) was also validated and conducted. GC analyses were carried out, using the gas chromatograph Varian 3300 (Varian, Walnut Creek, CA, U.S.A.), equipped with a fused silica capillary column DB-5 (30 m × 0.25 mm I. D., film thickness 0.25 μm) and a flame ionization detector (FID), where hydrogen (30 mL/min) in air (300 mL/min) was used. The column temperature program was 50 °C for 3 min, at a gradient of 8 °C/min, and upper isotherm of 260 °C for 5 min; the injection port and detector temperature was 260 °C, at a split ratio of 1:20, with carrier gas nitrogen (flow 1 mL/min). The test samples were dissolved in isooctane, and to all samples, nonane was added as an internal standard. Samples were mixed according to the following scheme: 1000 µL of isooctane + 10 µL of diesel fuel + 10 µL of nonane. Diluted samples with a suitable solvent, in this case, isooctane, improved the separation efficiency of the chromatographic column.

All measurements were conducted according to the valid standards; a list of them is provided in Table 3.

All parameters were always measured three times, and the results represent the average value from three measurements with the expanded uncertainty with a 95% confidence interval. The expanded uncertainty *U* of the measurand was obtained by multiplying the combined standard uncertainty *u*(*y*) by a coverage factor *k*, which provides the best estimate of the value attributable to the measurand. The value of the coverage factor *k* was chosen to meet the probability of coverage of about 95%, which, for a normal distribution, corresponds to the factor *k* = 2 [71]. 

Matlab 2015b (MathWorks, Natick, MA, USA) and R 4.0.3 (R Core Team) [72] were used for statistical evaluation and graphical representation of the results. Star Chromatography Workstation vs. 4.51 software (Varian, Walnut Creek, CA, USA) was used for GC data collection. 

## 3. Results

### 3.1. Fuel Parameters

The cetane number determines the ability of diesel fuel to ignite during compression ignition. Increasing the cetane number shortens the length of the ignition lag time. Decreasing the cetane number results in erratic engine operation and higher noise levels and has a negative effect on emissions, especially during cold starts when the engine does not generate sufficient heat to burn through the entire fuel charge. The result is increased CO and unburned and partially oxidized hydrocarbons—black smoke. The minimum cold start cetane number limit is 40 units; in the future, it is expected that the cetane number should be at least 56 units. Measurements are made on special diesel measurement engines, where a specific fuel sample is compared with a reference sample, and the ignition pattern is monitored to see if the ignition is the same in both cases when the compression ratio is changed. The reference samples used are cetane (n-hexadecane—C_16_H_34_) with a cetane number of 100 and 1-methylnaphthalene with a cetane number of 0.

Since the engine test is quite demanding for the cetane number measurement, a cetane index (CI) was later introduced to describe the ignition ability of fuel. The cetane index is determined based on the density (ρ) at 15 °C and distillation (temperature values of 10%, 50% and 90% recovered—T_10_, T_50_, and T_90_), according to the Equation (1) [63]. The cetane index does not come out the same as the cetane number for the same fuel; in practice, it is always several units lower.

(1) $$\begin{array}{c} CI=45.2+0.0892T_{10N}+\left(0.131+0.901B\right)T_{50N}+\left(0.0523-0.420B\right)T_{90N}\\ +0.00049\left(T_{10N}^{2}-T_{90N}^{2}\right)+107B+60B^{2} \end{array}$$

where *T*_10_*_N_* = *T*_10_ − 215, *T*_50_*_N_* = *T*_50_ − 260, *T*_90_*_N_* = *T*_90_ − 310, *B* = [*e*^−3.5^^(ρ − 0.85)^] − 1

The most important advantage of alcohol-based blends is that they can be used in diesel engines without any modifications. On the other hand, alcohols have a low cetane number, so their addition reduces the overall cetane number of the mixture. Ethanol has a cetane number of 5–8, and butanol, 12. The cetane number can be increased by the correct choice of the base diesel fuel at the refinery or by adding cetane booster additives, such as 2-ethylhexyl nitrate or 2,2-dinitropropane [73,74].

**Figure 3:**
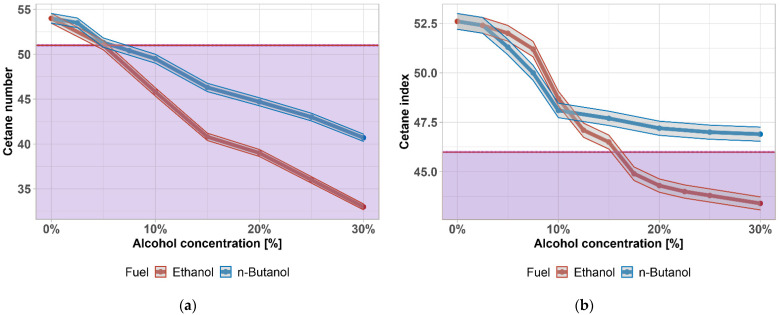
(**a**) Cetane number of diesel fuel–alcohol mixtures; (**b**) cetane index of diesel fuel–alcohol mixtures. Purple area highlights exceeding the limit given by the standard.

Figure 3a,b shows the change in the cetane number and cetane index as a function of the amount of butanol and ethanol added. The minimum permissible cetane number, according to EN 590, is 51, and the minimum cetane index, according to the same standard, is 46, both shown in purple. The grey area represents a measurement accuracy of ±0.76%. The addition of alcohol to diesel fuel results in a significantly lower cetane number. When 5% vol. ethanol or butanol is added to the diesel fuel tested, the cetane number of the fuel tested is already at the limit defined by the standard. There is a statistically significant difference between the cetane number and cetane index (*p*-value = 0.01053, resp. 0.0323); the data are normally distributed (*p*-value = 0.59, resp. 0.125). Similar results were found also in [75,76].

**Figure 4:**
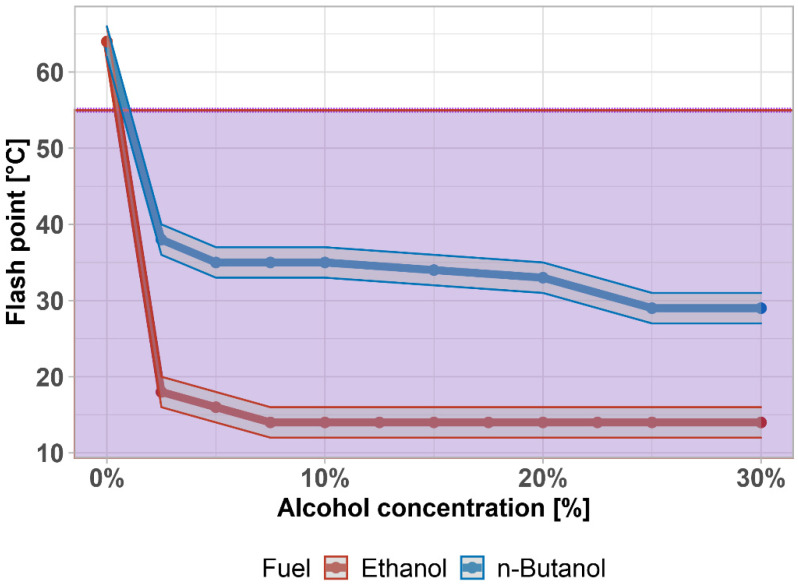
Flash point of diesel fuel–alcohol mixtures. Purple area highlights exceeding the limit given by the standard.

One of the most frequently monitored quality parameters is also the flash point. In general, it ranks flammable liquids into hazard classes. The minimum admissible value of the flash point of a diesel fuel is 55 °C, which characterizes it as Class II—shown by the purple area. The values of the flash point of pure diesel fuel are usually between 58 °C and 75 °C. The results in Figure 4 show (with ±1 °C accuracy) that the addition of butanol has a strong decreasing influence on the flash point, depending solely on its content in the diesel fuel. As results show, its value is below the minimum requirement of the EN 590 standard, even at 2.5 vol%. At higher concentrations of butanol, the value corresponds to the flash point of pure butanol. In comparison with ethanol, the temperature drop is even greater, and the mixture does not comply with EN 590. Without any other treatment, n-butanol–diesel fuel mixtures can be classified as Class I-C or Class II, whereas an ethanol–diesel fuel mixture would be classified as Class I-B or Class I-C. The flash point can be increased by the addition of terpineol [77]. This value, however, does not affect the combustion properties within the engine. It affects necessary safety measures related to the manipulation with a fuel. There is a statistically significant difference between flash points (*p*-value = 0.01379); the data are not normally distributed, so a non-parametric test was used (*p*-value = 0.000622). The same trend of a significant drop right after the first addition of light alcohol was also found by [78,79,80].

The cold filter plugging point (CFPP) is the temperature at which a layer of solidified paraffin forms a layer so thick that the liquid portion of the diesel no longer passes through the fuel filter sufficiently. When this temperature is reached, although the diesel is pumpable, the engine will shut down. Paraffins (a mixture of n-alkanes) are solids that are normally dissolved in the diesel, but as the temperature drops, they begin to release from the mixture and crystallize back into a solid, making it impossible to transport fuel to the engine. The CFPP temperature is the most important cold-flow parameter and roughly determines the temperature to which the diesel is usable. 

The CFPP is measured by the defined cooling of a diesel fuel sample in an apparatus where the diesel fuel is periodically passed through a system of fine sieves. Crystallized paraffins gradually clog the sieves and increase the pressure difference in front of and behind the sieves. The temperature at which a given pressure difference is reached is the temperature of the CFPP. The cold parameters differentiate the types of diesel fuel and are critical to the use and serviceability of diesel fuel in winter and in arctic climate zones. 

**Figure 5:**
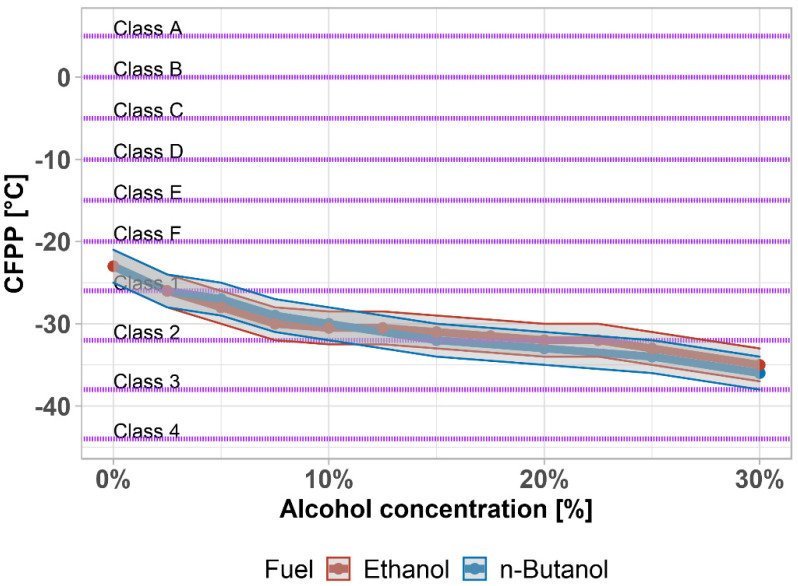
Cold filter plug point of diesel fuel–alcohol mixtures. Purple lines highlight the limit given by the standard.

The addition of ethanol or n-butanol to diesel fuel has a positive effect on the CFPP as can be seen in Figure 5 together with the ±1 °C accuracy. The CFPP gradually decreases from Class F to Class 1 (around 2.5%) and Class 2 (around 20%). This means that the fuel is usable, even in arctic climatic zones. There is no statistically significant difference between CFPP (*p*-value = 0.5271); the data are not normally distributed, so a non-parametric test was used (*p*-value = 0.0346). The CFPP was also measured by [78] with ~14% lower values, due to the use of arctic diesel fuel as a base.

Density is mainly determined by the aromatic content. It influences the calorific value of the fuel, which is related to the composition and proportion of each hydrocarbon, and it has also commercial importance in fuel supply, where it is used for conversions (mass–volume). The effect of the diesel fuel density on engine performance is due to the fact that the injection pump operates by volume, and therefore, the amount of fuel injected increases with the density. The specific fuel consumption decreases with increasing density. If the density of the diesel fuel is around the lower limit of the standard, there is a risk of damage to the moving parts of the fuel system (together with the lubricity). A lubricating film does not form on the moving parts, and excessive wear occurs. On the other hand, at high density, the mixture formation is impaired, due to insufficient fuel atomization—fuel droplets burn only on the surface, resulting in imperfect combustion. The share of unburned hydrocarbons, soot, and carbon monoxide in emissions then increases, which is reflected during acceleration and in full power mode as increased engine smokiness (black smoke). In addition, the density is also used to calculate the cetane index and can be used to infer the approximate composition of the diesel. 

**Figure 6:**
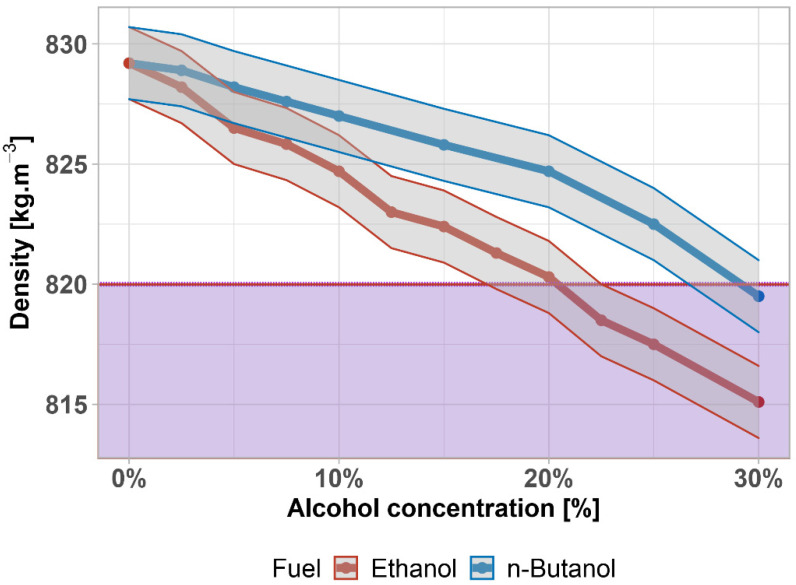
Density of diesel fuel–alcohol mixtures. Purple area highlights exceeding the limit given by the standard.

In the case of the density of alcohol–diesel fuel mixtures, there is no rapid decrease with the addition of n-butanol or ethanol (see Figure 6) together with the ±0.5 kg·m^−3^ accuracy. According to the EN 590, the lowest admissible density is 820 kg·m^−3^ at 15 °C—shown by the purple area. The requirement is met up to the concentration of 20 vol% of ethanol and 25 vol% of n-butanol. There is a statistically significant difference between densities (*p*-value = 0.00206); the data are normally distributed (*p*-value = 0.59). The density of butanol–diesel fuel mixtures was also investigated by [78], who found almost identical results (±1%).

The kinematic viscosity is a measure of the fluidity of diesel fuel and has some influence on its lubricity (as does density). Diesel with low viscosity does not adhere to the moving parts of the fuel system, reducing lubricity and increasing wear and risk of seizure. Viscosity has a significant effect on the droplet size of the fuel injected into the cylinder. Low viscosity has a positive effect on aerosol formation during diesel fuel injection into the combustion chamber. High viscosity causes imperfect fuel dispersion in the cylinder and can also lead to impaired diesel pumpability and impaired filter passage. 

**Figure 7:**
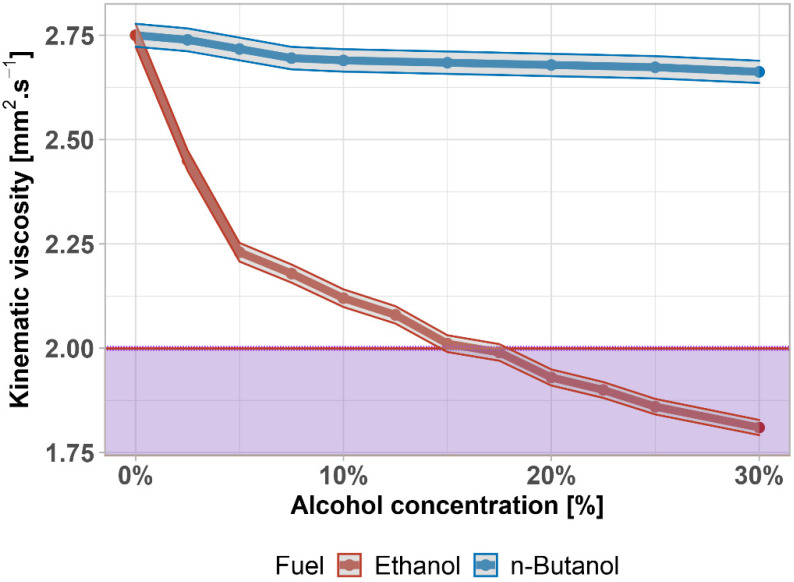
Kinematic viscosity of diesel fuel–alcohol mixtures. Purple area highlights exceeding the limit given by the standard.

According to the results of kinematic viscosity depicted in Figure 7, the influence of ethanol is very significant, whereas the influence of butanol is much lower. The requirements of EN 590 set the limits between 2.0 and 4.5 mm^2^∙s^−1^ (the lower limit is shown by the purple area). The viscosity of the n-butanol–diesel fuel mixture meets the limit in the whole tested interval. On the other hand, the addition of ethanol exceeds the limit by around 17.5%. Under this limit, there is a risk of damaging the moving parts of the fuel system, due to loss of the lubricating layer. There is a statistically significant difference between viscosities (*p*-value = 3 × 10^−4^); the data are normally distributed (*p*-value = 0.410). In comparison to [76,78], kinematic viscosity has same slowly declining trend.

Lubricity is an important property of diesel fuel, which is necessary to ensure the proper functioning of fuel pumps and injectors. 

The standard defines the minimum lubricity of diesel fuel as the diameter of the abrasion area, which is created by the friction of a vibrating ball on a metal surface. It is carried out in a special apparatus (high frequency reciprocating rig—HFRR) with the diesel fuel at 60 °C. The better the lubricity of the diesel, the smaller the friction area produced. In modern, sulfur-free diesel, it is increased by an additive, which is simpler than material or design modification.

**Figure 8:**
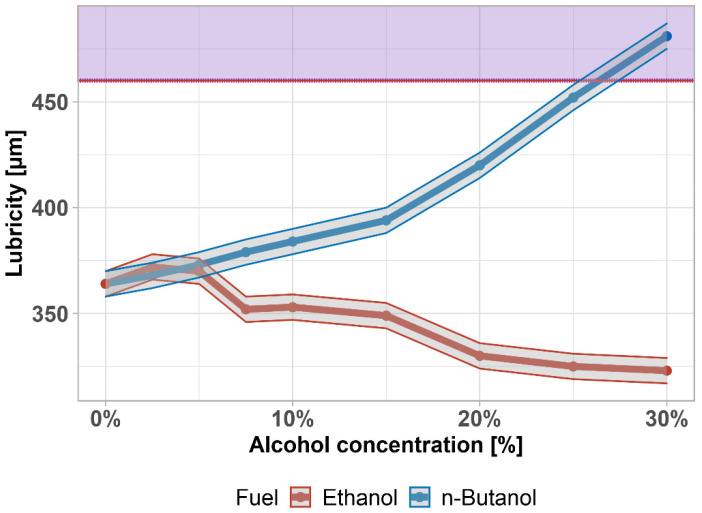
Lubricity of diesel fuel–alcohol mixtures. Purple area highlights exceeding the limit given by the standard.

According to the standard, the maximum permissible area diameter is 460 µm—shown by the purple area. This limit was exceeded above 25 vol% of n-butanol in diesel fuel (shown in Figure 8 together with the ±1% accuracy). Ethanol keeps the lubricity in almost the same values, much more than n-butanol. Since a lubricating layer is formed on the moving parts, there is no excessive wear. From this point of view, the addition of butanol does not represent any risk. Furthermore, this parameter can also be adjusted with suitable additives. There is a statistically significant difference between lubricities (*p*-value = 0.02689); the data are normally distributed (*p*-value = 0.137). 

Almost identical results in butanol–diesel fuel lubricity were found by Kuszewski, (±4%) [78]. A decrease in ethanol–diesel fuel lubricity was found also by Kuszewski et al. [81].

A summary of the physicochemical properties of mixtures that still meet the standard is given in Table 4.

**Figure 9:**
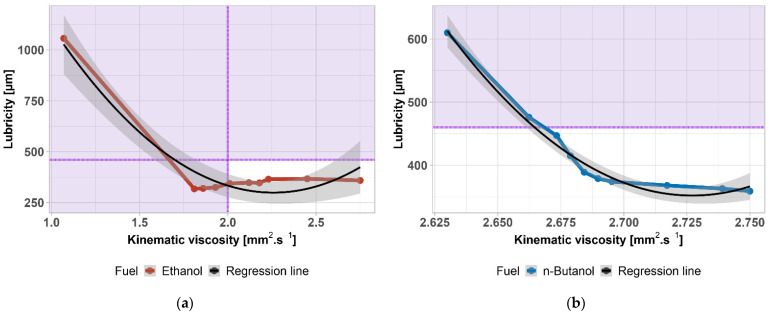
(**a**) Regression function of ethanol–diesel fuel lubricity as a function of kinematic viscosity; (**b**) regression function of n-butanol–diesel fuel lubricity as a function of viscosity. Purple area highlights exceeding the limit given by the standard.

As stated, the lubricity of the fuel is, to some extent, dependent on the kinematic viscosity of the fuel and the extent to which the lubricating layer adheres to the lubricated surfaces. For confirmation of this claim, a statistical evaluation of the dependence of lubricity on kinematic viscosity was performed. In order to cover the whole range, the lubricity of pure substances (100% ethanol and n-butanol) was taken from Table 2. Results are shown in Figure 9a,b, and Table 5. The grey area is the 95% confidence interval. The purple area highlights exceeding the limit given by the standard. A strong correlation was found for both alcohols in the whole range of concentrations. Assumptions for linear regression were satisfied (Table 6). 

### 3.2. Distillation Properties

The determination of the distillation curve is a dominant test, which has to be performed when the quality of the diesel fuel is assessed. By constructing a distillation curve, a picture of the predominant fraction is obtained, and the presence of higher or lower boiling fractions can be determined. For the fuel to burn in the cylinder, it needs to be vaporized and mixed with air, i.e., sufficiently fine atomization of the fuel during injection (small droplets have a larger total surface area and a higher evaporation rate), but also, a certain proportion of easily evaporable components, which low-alcohol fuel fulfils. The composition of the fuel should be such that it evaporates sufficiently quickly after injection into the cylinder and, therefore, runs regularly. Diesel fuel has to be sufficiently volatile so that the entire volume of fuel injected is vaporized, ideally starting with the lightest fractions, and at the same time, regularly, so that combustion is uniform. Additionally, fuel should contain heavier components, which will evaporate during the compression stroke when the combustion chamber walls are cooled. If, on the other hand, the diesel fuel contains too many light components, there is a risk of damage to the moving parts of the fuel system. Such diesel has significantly impaired lubricity. A lubricating film does not form on the moving parts and excessive wear occurs, which is not the case with low-percentage diesel–alcohol mixtures. 

**Figure 10:**
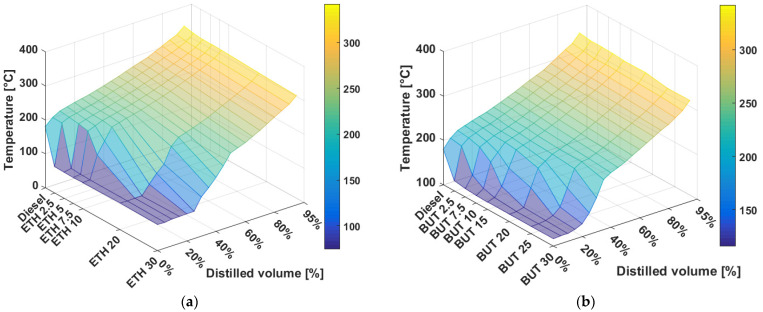
(**a**) Distillation curve of diesel fuel–ethanol mixtures; (**b**) distillation curve of diesel fuel–butanol mixtures.

It can be seen from Figure 10a,b that both ethanol and n-butanol significantly affect the beginning of the distillation curve. Mixtures up to 30 vol% of alcohol ensure the presence of heavier components contained in diesel fuel, which evaporate gradually during the compression stroke, during which the walls of the combustion chamber are cooled. The figures also show that after the distillation of alcohol, the distillation curve continues with the typical trend of diesel fuel distillation. 

The addition of alcohol in the diesel fuel will ensure a fine atomization of the fuel during injection, as the resulting droplets have a larger total surface area and a higher evaporation rate. Regarding the boiling point of butanol, it should not evaporate too quickly after injection into the cylinder, and thus, will not produce irregularity of the engine operation.

According to the BS EN 590+A1 standard, at 250 °C, the alcohol has to be distilled at less than 65 vol%, and at 350 °C at least 85 vol%; the temperature at 95 vol% distillation has to be at most 360 °C. This requirement is met for all fuels.

### 3.3. Gas Chromatography Analysis

There is no standard for the application of alcohols to diesel fuel that allows a more detailed analysis. Therefore, a simple analytical method for the determination of ethanol and butanol in diesel fuel, using GC-FID (gas chromatography with flame ionization detector), was developed and validated.

**Figure 11:**
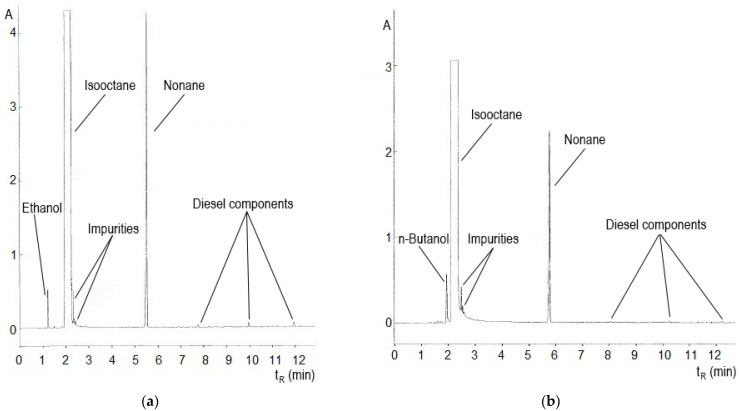
(**a**) Distillation curve of E10 mixture; (**b**) distillation curve of BUT10 mixture.

Gas chromatography can also provide a number of useful indicators about diesel fuel. In addition to information about the distillation profile and the content of individual compounds, it is possible to detect the presence of various impurities, or the presence of ethanol and n-butanol. In Figure 11a,b, chromatograms of 10 vol% mixture of ethanol–diesel fuel and n-butanol–diesel fuel are shown. The diesel components represent hydrocarbons C_14_, C_15_, and C_16_. All chromatograms were terminated after 12 min. In Table 7, precise concentrations of the fuel mixtures are given.

Deviations of the measured values from the reference values may be due to variations in the detector response or sorption of some components of the sample in the injection chamber. Variability of the measured values can be improved by using the auto-sampler and devices with electronic gas flow control.

## 4. Conclusions

Most of the literature deals mainly with the production process of biobutanol as a potential biofuel for internal combustion engines, but detailed testing of the properties of butanol blends are rather scarce and even more so for use in diesel engines and fuel standards.

With regard to their boiling points, both bioethanol and biobutanol affect the very beginning of the distillation curve of the blended fuel. However, blends of up to 30% alcohol by volume ensure the presence of the heavier components contained in diesel fuel for the proper combustion function of a diesel engine.

With the addition of bioethanol and biobutanol to diesel fuel, the density and viscosity are also reduced. Too low a density and viscosity can adversely affect the loss of the lubricating film necessary to lubricate the moving parts of the fuel system. Biobutanol has a higher viscosity, compared to both hydrocarbons and lower alcohols. While the drop in density corresponds to the density differences between diesel and alcohol, the effect of the hydrocarbon chain is clear from the viscosity drop, and biobutanol favors bioethanol in particular in this parameter. In terms of density, a maximum of 20% vol. bioethanol in the fuel and 25% vol. biobutanol is set as the limit, and in the case of viscosity, up to 15% vol. bioethanol, while biobutanol has almost no effect on the diesel viscosity parameter. 

The admixture of biobutanol in diesel fuel has a positive effect on the cold filter plug point (CFPP). Experiences with competing bioethanol have encountered difficulties with the miscibility of the two fuels and the stability of the blends at low temperatures as well as water binding, due to its hygroscopicity. However, there is no problem in the homogeneity of mixtures, even at very low temperatures when adding biobutanol to diesel fuel. Thus, biobutanol is also less corrosive to metal tanks and pipes, and the tested blends are completely stable at low temperatures, compared to bioethanol–diesel fuel. 

The addition of biobutanol to diesel also poses less risk of degradation of fuel lubricity, compared to bioethanol. Lubricity in diesel is also largely affected by changes in the kinematic viscosity parameters. However, diesel lubricity was not much exceeded in all tested blends (up to 30%) of biobutanol in diesel. In addition, this parameter can also be adjusted by using suitable additives (also by commercially available additives of biodiesel, where lubricity is above the measurement limit).

The admixture of both bioethanol and biobutanol also has a significant effect on the flash point of diesel fuel, which is categorized as a Class III flammable substance, according to EN 590. The addition of biobutanol at 2.5% and above can characterize the mixture as a hazard Class II combustible (the mixture with bioethanol is a hazard Class I combustible); however, even such a drop in the flash point does not affect the operation of the diesel engine.

## Figures and Tables

**Table 1:** Composition of various agricultural and other lignocellulosic residues [26].

Material	Cellulose (C_6_H_5_O_10_)_n_ [%]	Hemicellulose (C_5_H_8_O_4_)_n_ [%]	Lignin (C_31_H_34_O_11_)_n_ [%]
Algae (green)	20–40	20–50	—
Bagasse	32–48	19–24	23–32
Barley straw	31–45	27–38	14–19
Chemical pulp	60–80	20–30	2–10
Coir	36–43	0.15–0.25	41–45
Corn stalk	39–47	26–31	3–5
Corn stover	38–40	28	7–21
Cotton, flax	80–95	5–20	—
Grasses	25–40	25–50	10–30
Hardwood barks	22–40	20–38	30–55
Hardwoods	43–47	25–35	16–24
Newspaper	40–55	25–40	18–30
Rice straw	28–36	23–28	12–14
Softwood barks	18–38	15–33	30–60
Softwoods	40–44	25–29	25–31
Sorghum stalks	27	25	11
Sorghum straw	32	24	13
Sweet sorghum bagasse	34–45	18–28	14–22
Wheat straw	37–41	27–32	13–15

**Table 3:** Standards for the evaluation of the physicochemical properties.

Property	Standard
Diesel fuel	BS EN 590:2013+A1:2017 [61]
Cetane number	ISO 5165:2017 [62]
Cetane index	ISO 4264:2018 [63]
Density	ISO 3675:1998 [64]
Flash point	ISO 2719:2016 [65]
Kinematic viscosity	ISO 3104:1994 [66]
Lubricity	ISO 12156-1:2018 [67]
Cold filter plugging point	DIN EN 116 [68]
Distillation characteristics	ISO 3405:2011 [69]
Gas chromatography	EN 14078:2014 [70]

**Table 4:** Mixtures which comply with EN 590 + A1.

Mixture	Cetane Number	Cetane Index	Flash Point	Density	Kinematic Viscosity	Lubricity
Ethanol	≤5%	≤15%	<5%	≤20%	≤15%	>30%
n-Butanol	≤5%	>30%	<5%	≤25%	>30%	≤25%

**Table 5:** Parameters of the regression equation $\mathrm{WSD}=A+B\mu +{C\mu }^{2}$ depicted in Figure 9. Correlation coefficients, $R_{\mathrm{adj}}^{2}$, and *p*-value for both fuel mixtures.

Fuel	A	B	C	$R_{\mathrm{adj}}^{2}$	*p*-Value
Ethanol	2916.57	−2313.84	512.43	0.9238	5.071 × 10^−5^
n-Butanol	200,024	−146,405	26,838	0.9762	8.578 × 10^−7^

**Table 6:** Assumptions tests.

Dataset	Shapiro–Wilk Normality	Breusch–Pagan Heteroscedasticity
Ethanol	W = 0.8855	Χ^2^ = 0.1018
	*p*-value = 0.1508	*p*-value = 0.7496
n-Butanol	W = 0.9345	Χ^2^ = 0.3172
	*p*-value = 0.4939	*p*-value = 0.5733

**Table 7:** Measured concentrations of ethanol and n-butanol in diesel fuel.

Fuel	The Measured Concentration of Alcohol [vol%]
BUT 5	5.28
BUT 10	11.83
BUT 20	22.54
E10	10.72

## Data Availability

Data are contained within the article.
